# Video-Based Motion Capture Smartphone Apps for Testing Human Motor Performance Skills: Scoping Review

**DOI:** 10.2196/65474

**Published:** 2026-02-19

**Authors:** Clara Sophia Zoeller, Claudia Niessner, Manuel Fleps, Thorsten Klein, Anke Hanssen-Doose, Alexander Burchartz, Alexander Woll, Thorsten Stein

**Affiliations:** 1 Institute of Sports and Sports Science, Karlsruhe Institute of Technology, Engler-Bunte-Ring 15, Karlsruhe, 76131, Germany; 2 Institute of Movement and Sport, Karlsruhe University of Education, Karlsruhe, Germany

**Keywords:** smartphone app, motion capture, motor performance skills, motor assessment, automated video assessment, AI-based motion capture, kinematics, artificial intelligence, mobile health, scoping review, physical health, mental health, neurological illnesses, healthy adults, older, athletes

## Abstract

**Background:**

Good motor performance skills (MPS) are relevant in all stages of life. Higher MPS are associated with enhanced cognitive abilities and physical and mental health. The assessment of MPS is important to identify deficits in MPS at an early stage and to implement interventions to address these deficits. One method to assess MPS is through marker-based motion capture in a laboratory setting with multiple cameras. However, this approach is expensive and time-consuming, making it impractical, for example, in large-scale studies for MPS assessment. Recent advancements (eg, artificial intelligence) in technology (eg, smartphone cameras) have opened up innovative solutions for various challenges (eg, testing large sample sizes). A potential solution is using video-based smartphone apps to assess MPS through markerless motion capture with a single camera.

**Objective:**

The objectives of this scoping review were to summarize existing smartphone apps designed to digitally assess MPS through motion capture, identify the target population of the apps, determine whether the apps have been validated, and summarize the specific MPS that were assessed.

**Methods:**

The scoping review was conducted in accordance with the PRISMA-ScR (Preferred Reporting Items for Systematic Reviews and Meta-Analysis extension for Scoping Reviews) guidelines. The search was conducted in March 2024 using PubMed, Scopus, SPORTDiscus, Web of Science, Education Resources Information Centre, and SAGE Publications. All included studies investigated video-based motion capture smartphone apps to assess MPS.

**Results:**

A total of 10 studies met the inclusion criteria. Seven different smartphone apps were used within the studies, 6 of which have already been validated. The MPS assessed through the apps were gait, breaststroke, running, countermovement jump, and shoulder mobility, and 1 study assessed a functional movement test battery. The studied populations were healthy adults, older adults, athletes, or individuals with neurological illnesses.

**Conclusions:**

The assessment of MPS through smartphone apps represents a promising tool, which can be used in a variety of fields, such as health and performance monitoring, coaching, and scientific research. In the future, more studies should focus on developing new smartphone apps to assess different MPS and validate these apps.

## Introduction

The comprehensive development of motor performance skills (MPS) is critical for healthy human development [1] and remains important throughout all stages of life [2]. Overall, studies have shown that higher MPS are associated with increased physical activity and physical fitness at all ages [3-5], which have both physical and mental health benefits (eg, reduced depressive symptoms) [3,6]. Furthermore, there is an inverse relationship between MPS levels and body weight [5,7,8].

Given the multitude of terms used in motor performance research, it is important to select a definition of the term “motor performance skills” that will be used in this study. In their scoping review, Sortwell et al [9] established a general definition of the MPS term based on existing descriptions in the literature. MPS encompass a range of movements, including those used for space coverage (eg, walking), object manipulation (eg, throwing), and movements that are needed for overcoming obstacles in vertical, diagonal, or horizontal directions (eg, climbing and jumping). Additionally, they include different kinds of pulling, pushing, and holding. These MPS serve as the foundation for specialized movement and the acquisition of sport-specific competencies [9]. The term MPS is often used interchangeably with the terms “motor skills” and “fundamental movement skills” [9,10].

The use of motor performance test batteries to assess motor performance allows for a precise examination of the current state and development of MPS. The results of these test batteries can be used to target interventions aimed at reducing deficits and improving performance [1,11,12]. However, assessing MPS using test profiles only provides limited or mainly subjective information regarding execution errors or the quality of the movement performed. A quantitative biomechanical measurement of kinematic variables collected via motion tracking systems could serve as an alternative or complementary approach [13,14].

The current gold standard for biomechanical movement assessment is using marker-based 3D motion tracking systems in a laboratory environment [14,15]. However, this method is time-consuming, expensive, and complex to implement, limiting its application in large-scale population studies [15-17]. To address these limitations, some studies have validated markerless 2D motion capture systems by comparing them to the gold standard. For example, a recent study showed a very high agreement between the gold standard and a single smartphone camera in a countermovement jump analysis, where videos were evaluated by Sbsq-pose (Subsequent GmbH), an artificial intelligence (AI)–based motion tracking system using deep learning to predict the 3D positions of 24 skeletal key points from 2D video recordings [18]. Indeed, the use of AI has gained increasing attention in biomechanics [19]. Today, there are pose estimation models that can be used to analyze video recordings of human movement [19]. These pose estimation models are created by deep learning algorithms, which are capable of identifying anatomical landmarks from digital videos [13]. To ensure that the assigned anatomical landmarks are correctly recognized, these human body models are first trained on large datasets [13]. Human annotators manually label the positions of anatomical landmarks in the training images [13,20]. Subsequently, the AI algorithm is able to automatically track human movements within recorded videos [13]. The trained pose estimation models also enable motion capture to be integrated into various everyday apps, such as smartphone apps [13,19]. There are already several smartphone apps that provide biomechanical monitoring, such as gait analysis, which provides information on spatiotemporal parameters (eg, cadence and foot strike), joint kinematics (eg, knee flexion angle) but not joint kinetics [21]. These apps enable automatic kinematic detection of whole-body and partial-body movements, including motion analysis that can be used in the field with minimal effort and even with large sample sizes. The advantage of using smartphone apps to collect MPS is that it is a simple and cost-effective way to perform biomechanical studies on a large population without the need for a lot of equipment or a laboratory environment, as the majority of individuals possess a smartphone [13]. In 2022, 6.4 billion individuals worldwide owned a smartphone, with an estimated 6.9 billion in 2024 and an estimated total of 7.7 billion by 2028 [22]. The widespread ownership of smartphones enables testing of large numbers of individuals, which is not feasible with a laboratory-based approach. The use of smartphone apps may be a particularly beneficial approach for population-based fitness studies, such as the MoMo study [23,24] in Germany or as those using the ALPHA test battery, which includes highly reliable and valid measures of cardiorespiratory fitness, muscular strength, and anthropometry [23-25]. For instance, a large-scale fitness study in European youth successfully collected data from nearly 8 million test results across 34 countries, encompassing a broad age range and using representative national data [25]. By using smartphone apps, a similar number of participants could be assessed on their fitness levels more efficiently. This approach minimizes the need for extensive travel and logistical resources, thereby enabling greater flexibility in test scheduling. By allowing participants to complete assessments remotely from their homes, it eliminates the requirement for in-person appointments or visits to testing facilities, thus streamlining the data collection process and enhancing accessibility for a broader population. However, it is imperative to acknowledge the limitations inherent to such apps, which often exhibit performance deficiencies. A number of factors may contribute to inaccurate movement detection by smartphone cameras. These include, for example, a low sampling rate, the presence of human-like objects in the room, and inaccurate camera positioning. These issues can result in the inability to precisely detect anatomical orientation points, which, in turn, can lead to measurement errors [13]. Furthermore, it should be noted that certain apps are designed to operate within a cloud computing environment, necessitating a stable internet connection for optimal functionality. This aspect should be considered when using such s.

To date, there is one review that gives an overview of video-based motion capture smartphone apps for MPS testing, but it only focuses on healthy adults [26]. Currently, no reviews provide a general overview for all population types.

This gap in the literature hinders the ability of researchers and practitioners to fully understand the capabilities and limitations of these technologies. Therefore, we conducted a scoping review to address this gap by providing a comprehensive overview of the current state of research on existing smartphone apps designed to assess the biomechanics of MPS through markerless motion capture. This review aimed to highlight the advancements, identify potential areas for improvement, and guide future research and development in this field.

We specifically addressed four aims: (1) to summarize existing smartphone apps designed to digitally assess MPS through motion capture, (2) to elucidate the specific MPS that these apps assess, (3) to determine whether these apps have been validated, and (4) to identify the target population of each app.

## Methods

This scoping review was performed in accordance with the PRISMA-ScR (Preferred Reporting Items for Systematic Reviews and Meta-Analysis extension for Scoping Reviews) guidelines (Checklist 1) [27]. A study protocol was designed in advance and then registered on OSF [28].

### Eligibility Criteria

#### Inclusion Criteria

The studies included in the review had to perform motor performance tests that were assessed through video-based motion capture integrated into smartphone apps. The papers were included if they were written in English, published up to February 2024, involved human participants, and assessed motor performance through an AI-based video analysis (without human supervision).

#### Exclusion Criteria

All articles that used the smartphone’s built-in sensors or accelerometers for the assessment of MPS were excluded from the analysis. We also excluded dissertations and conference papers. Meta-analyses, scoping reviews, and systematic reviews were not included in this scoping review but were considered separately in this paper as part of the basis for discussion and can be found in the “Meta-Analyses and Reviews” section.

### Search Strategy

To identify potentially relevant articles, the search was conducted on March 5, 2024, in six electronic databases: PubMed, Scopus, SPORTDiscus, Web of Science, Education Resources Information Centre, and SAGE Publications. Before beginning the search, 2 reviewers (CSZ and CN) screened various publications and reviews in the research area, discussed the results, and determined the search term. The search terms included topics related to (1) MPS, (2) motion capture, and (3) smartphone apps, which were combined by Boolean operators. The search terms are shown in the Multimedia Appendix 1. The search was performed on the title and abstract of the papers, and the study type (validation study) was screened manually. There were no preset restrictions on the target population, so all ages were included. Before performing the search, the search strategy was reviewed by an expert (Dr Janis Fiedler) in mobile health technologies. Gray literature was not considered in this scoping review.

### Selection of Sources of Evidence

To ensure a consistent and transparent selection process, a standardized screening form was developed by 2 reviewers (CSZ and CN) based on the central research objective: to provide a comprehensive overview of smartphone apps that assess MPS through markerless motion capture. The form was piloted using a small set of studies to calibrate inclusion decisions and refine key criteria such as app type, motion capture method, and degree of automation.

### Study Selection

The search results were exported into the citation program Zotero (Corporation for Digital Scholarship). After removing duplicates, the titles and abstracts were screened by one reviewer (CSZ) using the open-source machine learning software “ASReview” (ASReview LAB developers) developed at Utrecht University [29]. To start training the model, 2 studies were initially selected for inclusion, and 2 studies were selected for exclusion [29]. Subsequently, the software presented 1 paper at a time, and the reviewer determined if it should be included or excluded based on its title and abstract. The title and abstract screenings were stopped after 50 consecutive articles were excluded by the reviewer [30,31]. Afterward, the second reviewer (CN) screened the articles excluded by the first reviewer to ensure that no relevant articles were falsely excluded. Following the title and abstract screening, the software provided a list of the included articles, which were then screened manually. If the full text was not available, authors were contacted and asked to provide it. The full-text screening was performed independently by 2 reviewers (CSZ and MF).

### Data Charting and Extraction

To chart the data, 2 reviewers created a data charting form in Microsoft Excel to determine which variables should be extracted. Data extraction was then performed by 1 reviewer (CSZ) and verified by another author (TK). During the screening process, we found that information on the validation of the apps was more relevant than initially anticipated. Therefore, a fourth research objective addressing this aspect was added. This refers to whether the app was compared with another measurement instrument, such as a marker-based motion tracking system, regardless of whether the app was validated in the included study or in a previous study. The variables listed in the data chart included information about the authors (first author and year of publication), study design, study aims, population (sample size, age, and gender), name of the smartphone app, assessed MPS, if the app has been validated, and the main findings of the studies. The studies were grouped according to the smartphone app and the MPS studied.

## Results

### Study Selection

**Figure 1.**
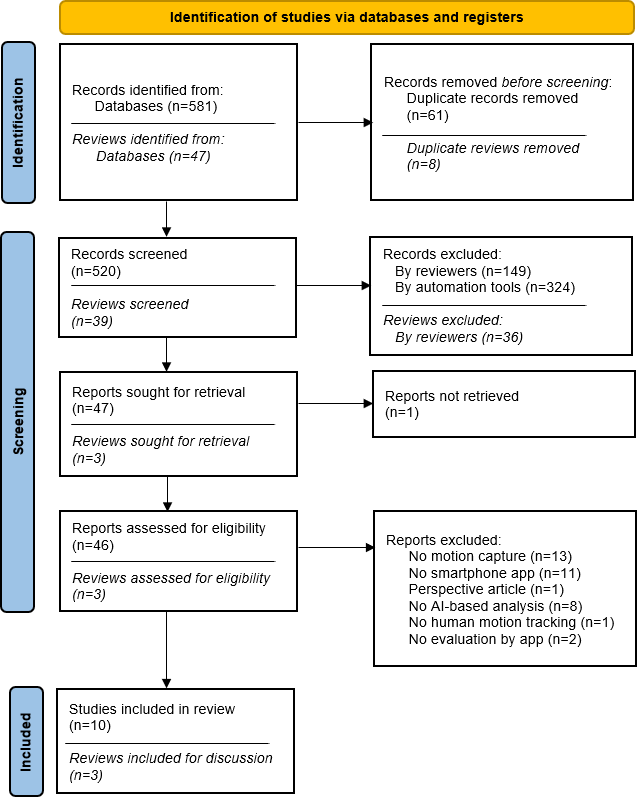
Flow diagram of search strategy. Italicized words refer to the selection of separately considered meta-analyses and reviews. AI: artificial intelligence.

A total of 581 articles were identified by searching the electronic databases. After removing duplicates, 520 (89.4 %) articles were included in the title and abstract screening in ASReview. The number of articles was subsequently reduced to 47 (8.1 %), with 1 article being excluded due to unavailability. Therefore, 46 (7.9 %) articles were included in the full-text screening. On the basis of the full-text screening, a total of 10 (1.7 %) articles were included in this scoping review. A detailed overview of the selection process is shown in Figure 1.

### Study Characteristics

The included articles were published between 2021 and 2024, with the most articles published in 2022 (n=3) and 2023 (n=4). Four of the studies were conducted in Japan [32-35]. The remaining studies were conducted in Australia [36], Germany [37], Spain [38], the United Kingdom [21], and the United States [39]. One study did not indicate the country in which it was conducted [40]. Research designs of the studies were mainly validation studies (n=5) [21,32,36,37,39], 1 proof-of-concept study [38], 2 cross-sectional studies [34,40], and 2 prospective, observational studies [33,35] (Table 1).

**Table 1.** Study characteristics.

Study	Study type	Participants: sample size; sex (male or female); age (y), mean (SD)	Smartphone app; pose estimation algorithm	MPS^a^	Validated
Aoyagi et al [32]	Validation study	N/A^b^	TDPT-GT^c^; modified Resnet34 image classification model	Gait	Yes^d^
Azhand et al [37]	Validation study	N=44 healthy older adults; N/A; 73.9 (6.0)	N/A; series of algorithms: Convolutional Pose Machine 25 body joint model, VNect model, and GASTNet 3D uplifting model	Gait	Yes
Balsalobre-Fernández [38]	Proof-of-concept case study and validation study	N=1 healthy physically active man; n=1 male and n=0 female; 36	My Jump Lab; Apple Vision framework	CMJ^e^	Yes
Fanton et al [39]	Validation study	N=150 (n=113 healthy adults, n=17 athletes, and n=20 MI^f^ adults); n=56 males and n=94 females; 18-85	Amazon Halo Movement; N/A	Single leg stance, forward lunge, overhead squat, feet together squat, overhead reach	Yes
Feng et al [40]	Cross-sectional study	N=8 professional swimmers; n=8 males and n=0 females; 21.8 (2.6)	N/A; series of algorithms: Gaussian filtering, Canny operator, double threshold method, and Hough transformation	Breaststroke	No
Iseki et al [35]	Prospective and observational study	N=23 patients with PD^g^; n=13 males and n=10 females; 70.1 (6.0) N=23 patients with iNPH^h^; n=16 males and n=7 females; 77.0 (6.4) N=92 healthy adults; n=36 males and n=56 females; 72.3 (6.3)	TDPT-GT; Modified Resnet34 image classification model	Gait	Yes^d^
Iseki et al [34]	Cross-sectional study	N=114 pathological patients (n=48 iNPH, n=21 PD, and n=55 other NMD^i^); n=52 males and n=62 females; 74.5 (7.8) N=160 healthy adults; n=91 males and n=69 females; 72.9 (11.1)	TDPT-GT; Modified Resnet34 image classification model	Gait	Yes^d^
van den Hoorn et al [36]	Validation study	N=20 healthy adults; n=10 males and n=10 females; 36 (13)	mymobility App; Apple Vision framework	Shoulder abduction, adduction, flexion, and extension	Yes
Yamada et al [33]	Prospective and observational study	N=15 healthy adults; n=9 males and n=6 females; 39.1 (20.1) N=92 older adults; n=36 males and n=56 females; 73.0 (6.3) N=47 patients with iNPH; n=32 males and n=15 females; 77.3 (6.3)	TDPT-GT; Modified Resnet34 image classification model	Gait	Yes^d^
Young et al [21]	Validation study	N=31 healthy, experienced runners; n=20 males and n=11 females; 34.5 (9.7)	N/A; BlazePose	Running	Yes

a MPS: motor performance skills.

b N/A: not applicable.

c TDPT-GT: Three-Dimensional Pose Tracker for Gait Test.

d Preliminary validation indicates an initial comparison with another.

e CMJ: countermovement jump.

f MI: movement impaired.

g PD: Parkinson disease.

h iNPH: idiopathic normal pressure.

i NMD: neuromuscular diseases.

### MPS and Smartphone Apps

The following results are presented in accordance with the initial 3 aims of this review and are summarized descriptively to provide an overview of the identified studies (Table 1, Multimedia Appendix 2).

#### Gait

In the studies included in this review, gait was the most commonly assessed MPS (n=5). Four of the studies used the Three-Dimensional Pose Tracker for Gait Test (TDPT-GT) smartphone app to assess this MPS [32-35]. Aoyagi et al [32] developed the TDPT-GT app to quantitatively assess pathological gait patterns and performed a preliminary validation against a Vicon 3D motion tracking system. The app uses a modified ResNet34 image classification model to detect the coordinates of 24 anatomical key points and calculate the 3D angles of the lumbar, bilateral hip, neck, knee, and ankle joints. To conduct the test, the subjects were instructed to walk in a circle with a diameter of 1 m, both clockwise and counterclockwise [32-35]. The app is capable of reconstructing 3D full-body movements from 2D motion capture data [32].

The other 3 studies examined the gait patterns of idiopathic normal pressure hydrocephalus (iNPH), Parkinson disease, and other neuromuscular diseases, as well as older adults and healthy adults [33-35]. Two studies by Iseki et al [34,35] compared pathological gait patterns with healthy gait patterns.

The final study included in the review used the TDPT-GT app to determine distinct indices for three pathological gait patterns: shuffling gait, wide-based gait, and short-stepped gait [33].

The fifth study developed a new smartphone app for gait assessment and examined the validity and repeatability of the app (the app name was not mentioned) [37]. To test the validity of the smartphone app, a comparison with the GAITRite System was performed. The app includes a series of pose estimation algorithms starting with the Convolutional Pose Machine 25 body joint model, followed by the VNect model and the GASTNet 3D uplifting model, to extract four gait parameters: gait speed, step length, step time, and cadence.

#### Running

One study developed an unnamed smartphone app that uses the deep learning pose estimation tool BlazePose (MediaPipe framework, Google LLC) to assess running gait in experienced runners [21]. To validate the app, the researchers simultaneously recorded the participants running on a treadmill with an iPhone 13 smartphone camera and a Vicon 3D motion tracking system [21].

#### Countermovement Jump

The study of Balsalobre-Fernández [38] used the Apple Vision framework (Apple Inc.), integrated into the My Jump Lab app, to assess countermovement jump (CMJ) height. The app was validated by comparing the results from My Jump Lab to those recorded simultaneously using a force plate.

#### Shoulder Mobility

One study performed shoulder range of motion tests by using the mymobility app, which uses the Apple Vision framework (Apple Inc.) for pose estimation [36]. For validation, participants performed shoulder abduction, adduction, flexion, and extension while being filmed with an iPhone 13s camera, with a sample rate of 30 frames per second, and a marker-based Vicon motion tracking system for comparison.

#### Functional Movement Test Battery

The study of Fanton et al [39] validated the Amazon Halo Movement app (Amazon Ltd) by comparing it to validated functional movement tests (FMTs) and sensor-collected kinematic variables. The FMT battery of the smartphone app was performed by participants wearing a full-body suit equipped with motion sensors. The app includes 5 FMTs, which are used to assess posture, stability, and mobility (single leg balance, forward lunge, overhead reach, feet together squat, and overhead squat) [39].

#### Breaststroke

The study of Feng et al [40] used a nonvalidated smartphone app to assess breaststroke technique. The app records the swimmer’s movement in the water through a smartphone camera and calculates the angles between the thigh and trunk, thigh and calf, and the trunk and the horizontal plane. The video processing integrated into the app starts with Gaussian filtering, followed by the calculation of gradient amplitude and direction with a Canny operator, double threshold method for edge closure, and, finally, the detection of a straight line by Hough transformation [40].

### Population

Most studies used the apps to assess the MPS of healthy adults (n=6) [33,35,36,38,39], 3 studies included athletes (general athletes [39], swimmers [40], and runners [21]), 2 studies included older persons [33,37], and 4 studies included clinical populations, that is, Parkinson disease [34,35], iNPH [33-35], movement impairment [39], and other neuromuscular diseases [34]. One of the studies did not provide any information about their study population [32] (Multimedia Appendix 3).

### Synthesis of the Findings

The included studies demonstrate a growing interest in smartphone-based digital assessment tools for MPS, with gait analysis being the most frequently targeted skill, especially in clinical contexts such as Parkinson disease and iNPH (Multimedia Appendix 4). Despite the variety of apps, most tools have been validated through intraclass correlation coefficient or gold standard comparisons such as marker-based 3D motion capture. Deep learning algorithms, particularly modified ResNet34, dominate technical implementation. However, there are clear research gaps: few studies address upper body skills, and most studies focus on adults in controlled settings rather than broader, general-use or preventive contexts. These insights highlight future needs for diversified and validated apps, including those for different target groups (eg, children), and increased attention to real-world usability and accessibility (Table 2).

**Table 2.** Overview table of key findings.

Category and subcategory	Studies, n (%)	Validated, n (%)	Example applications or notes	Studies included
Motor performance skill				
Gait	5 (50)	5 (50)	TDPT-GT^a^, CPM^b^+VNect	Aoyagi et al [32]; Azhand et al [37]; Iseki et al [34,35]; Yamada et al [33]
Running	1 (10)	1 (10)	BlazePose	Young et al [21]
CMJ^c^	1 (10)	1 (10)	My Jump Lab	Balsalobre-Fernández [38]
Shoulder mobility	1 (10)	1 (10)	mymobility	van den Hoorn et al [36]
Functional movement battery	1 (10)	1 (10)	Amazon Halo Movement	Fanton et al [39]
Swimming (breaststroke)	1 (10)	—^d^	Series of algorithms: Gaussian filtering, Canny operator, double threshold method, Hough transformation	Feng et al [40]
Target population				
Healthy adults	6 (60)	—	Most common group	Balsalobre-Fernández [38]; van den Hoorn et al [36]; Young et al [21]; Azhand et al [37]; Iseki et al [34,35]; Yamada et al [33]
Athletes	3 (30)	—	Swimmers, runners, general athletes	Young et al [21]; Feng et al [40]; Fanton et al [39]
Older adults	2 (20)	—	Often in gait-related studies	Azhand et al [37]; Yamada et al [33]
Clinical populations	4 (40)	—	PD^e^, iNPH^f^, MI^g^, NMD^h^	Iseki et al [34,35]; Yamada et al [33]; Fanton et al [39]
Validation methods				
ICC^i^	6 (60)	—	My Jump Lab, BlazePose, CPM-based apps	Azhand et al [37]; Balsalobre-Fernández [38]; van den Hoorn et al [36]; Young et al [21]; Fanton et al [39]
AUC^j^	3 (30)	—	TDPT-GT for neurological assessment	Iseki et al [34,35]; Yamada et al [33]
Motion capture comparison	4 (40)	—	Vicon system	Balsalobre-Fernández [38]; van den Hoorn et al [36]; Young et al [21]; Azhand et al [37]
No formal validation	1 (10)	—	Swimming app	Feng et al [40]
Algorithms and frameworks				
Modified ResNet34	4 (40)	—	Clinical gait tracking (TDPT-GT)	Aoyagi et al [32]; Iseki et al [34,35]; Yamada et al [33]
Apple Vision Framework	2 (20)	—	My Jump Lab, mymobility	Balsalobre-Fernández [38]; van den Hoorn et al [36]
BlazePose	1 (10)	—	Running gait analysis	Young et al [21]
CPM+VNect+GASTNet	1 (10)	—	Multistep pose extraction for gait	Azhand et al [37]
Algorithm series	1 (10)	—	Hough/Canny in swimming	Feng et al [40]

a TDPT-GT: Three-Dimensional Pose Tracker for Gait Test.

b CPM: convolutional pose machine.

c CMJ: countermovement jump.

d Not applicable.

e PD: Parkinson disease.

f iNPH: idiopathic normal pressure hydrocephalus.

g MI: movement impairment.

h NMD: neuromuscular disease.

i ICC: intraclass correlation.

j AUC: area under the curve.

### Meta-Analyses and Reviews

#### Selection of Meta-Analyses and Reviews

As meta-analyses, scoping reviews, and systematic reviews were not included in the scoping review, they were excluded from the screening process conducted by ASReview and were screened manually (CSZ and CN). A detailed overview of the screening is also found in Figure 1, shown in italics. To gain a more comprehensive understanding of the research field and to provide a basis for discussion, the inclusion criteria were considered more generously here. As a result, reviews were included that consider pose estimation methods without apps or apps that require a human operator to assess MPS.

#### Findings From Meta-Analyses and Reviews

We did not find any meta-analyses, reviews, or similar studies analyzing video-based motion capture smartphone apps. Using our search terms, we identified 47 meta-analyses and reviews, of which 3 have been included. However, one of the reviews included studies that examined apps that require manual operation, while the others included studies on pose estimation but without the use of smartphone apps. The 3 reviews present the respective findings relevant to specific subareas of video-based motion capture smartphone apps, which justified their inclusion in the evaluation.

The systematic review by Silva et al [41] focused on smartphone apps for capturing strength, power, change of direction, and velocity assessment. The included studies analyzed a total of 11 different smartphone apps, but 10 of them required a human operator to manually perform the assessments. Only the included study by Balsalobre-Fernández et al [42] used a video-based motion capture app (My Lift app). However, the app focused on tracking the barbell used during the snatch motion, rather than on the human movement itself. Nonetheless, this review provides valuable insights for an overview of the research and supports our aim of identifying existing apps, even if most of them require manual operation.

Furthermore, the review by Stenum et al [13] provides a general overview of the research on AI-based human pose estimation and its application in monitoring MPS, but without the use of smartphone apps. This review presents the extensive range of applications for these estimation tools, which are used in monitoring human development, in the motor assessment of neurological diseases, and in performance optimization. The advantages of these tools include a cost-effective and widely accessible alternative to motion capture systems in the laboratory [13].

In light of the growing use of pose estimation software in AI-based smartphone apps, the review by Stenum et al [13] was included to provide a more comprehensive understanding of the field.

Another review examined various technologies for markerless motion capture in combination with pose estimation in clinical surveys [43]. The review included a total of 65 studies, which, in turn, identified 9 different technical devices for markerless motion capture. The 3 most commonly used technologies were the Microsoft Kinect system, cameras such as GoPro, and smartphone video recordings. The review offers a comprehensive overview of the current state of research in this field and highlights the potential of video-based motion capture as a screening tool [43].

## Discussion

### Principal Findings

Video-based motion capture smartphone apps enable automatic kinematic detection of whole-body and partial-body movements, including motion analysis that can be used in the field with minimal effort and even with large sample sizes. To provide an overview of the current state of research on such smartphone apps, the scoping review had four aims: (1) to summarize existing smartphone apps designed to digitally assess MPS through motion capture, (2) to elucidate the specific MPS that these apps assess, (3) to determine whether these apps have been validated, and (4) to identify the target population of each app.

The findings of this scoping review indicate that there is a variety of smartphone apps that enable the recording and evaluation of MPS through video-based motion capture. Here, “gait” was the most frequently assessed MPS in the included studies, which was mainly recorded using the TDPT-GT app [32-35]. A total of 7 different smartphone apps were used in the aforementioned studies. Six of these apps have already been validated by comparing them with the corresponding gold standard methods. The smartphone apps identified here only include healthy adults, older adults, and individuals with neurological diseases as the target population. Children and adolescents have not yet been the focus of such smartphone apps, but it would be important to develop apps for this target group, for example, to identify motor development disorders in this target group at an early stage to provide appropriate interventions.

As demonstrated by the publication years of the articles, the research field of automatically assessing MPS with the use of video-based smartphone apps is a relatively novel and expanding area. The first publications in this field appeared in 2021 [37].

There are several advantages to assessing MPS through video-based motion capture smartphone apps. One advantage is that it represents a more cost-effective alternative to laboratory-based systems, such as marker-based 3D motion capture or direct observation by trained professionals and offers the advantage that it can be performed in any location [14,16,17]. Furthermore, the pose estimation software integrated into the app does not require the attachment of markers on the participant, making it less time-consuming than marker-based motion tracking systems [14,17]. Additionally, some groups of people, such as older adults or those with disabilities, may be unable to tolerate the lengthy process of marker attachment and subsequent measurement [35].

These apps offer versatile use in coaching, health and performance monitoring, and scientific research. In scientific research, they can be used to perform testing outside of the laboratory setting and when no expensive motion capture system is available [13]. In the field of health monitoring, these applications would enable the testing of patients at home, as exemplified by the TDPT-GT app [32-35] included in this review. The advantage of testing at home is that patients are not required to visit clinics, physicians’ offices, or physiotherapists as frequently [44]. Furthermore, these smartphone apps enable patients to be screened more frequently, which, on the one hand, relieves the burden on physicians and, on the other hand, allows the identification of developmental disorders or other impairments at an earlier stage, thus enabling timely intervention [13]. Additionally, it enables patients to be screened in their everyday environment, which is of great relevance depending on the clinical picture [44]. However, it must be considered that there is a nonnegligible challenge in the use of smartphone apps in epidemiologic health studies, which is the consideration of data sensitivity and privacy issues [45]. These applications often collect and process sensitive health data, which must be protected to ensure participants’ privacy. Robust data encryption, secure data storage, and strict access controls are essential to safeguard this information. Furthermore, clear communication with participants about data usage, consent, and their rights is imperative to maintain trust and compliance with data protection regulations. Ensuring these measures are in place will facilitate the ethical and effective use of smartphone apps in health research [45].

In addition to the frequent screening of patients with impairments, the applications can also be used to monitor the athletic performance, as demonstrated in the study of Feng et al [40]. The advantage of the used pose estimation software integrated into the apps is that it enables real-time motion tracking and therefore can provide real-time feedback [13]. This is a valuable addition to the feedback provided by the coach, and it allows for a more frequent performance monitoring, as it enables monitoring during training sessions when the coach is not present. The potential for real-time motion tracking and feedback represents a valuable addition to the field of health monitoring. To ensure these apps deliver reliable and valid results, further development and validation against gold standard methods are essential [13].

Another advantage of using smartphone apps that have integrated AI for pose estimation is that they are less prone to inaccuracies than apps that require an operator to estimate, for example, the start and stop frames of a movement and then set these events manually [41,44].

As the technological revolution progresses, the potential of smartphone apps in scientific research, health monitoring, and athletic performance becomes increasingly evident.

### Limitations

Despite the benefits of pose estimation, the following limitations must be considered when using these algorithms. For example, wearing loose clothing, such as dresses, can cause anatomical landmarks to be misidentified or obscured by other parts of the body. Environmental factors, such as lighting conditions, background clutter, or camera positioning, can also significantly affect the accuracy of pose estimation [46]. Variability in these external factors may introduce inconsistencies in results, especially in field-based assessments where standardization is difficult to maintain. It should also be noted that cameras with low sampling rates may not be able to correctly capture fast movements or that the training data used to train the algorithms may be very different from the movements to be analyzed and therefore be prone to error [13]. Another salient problem concerning the training data is that the annotators may lack anatomical knowledge, which can compromise their ability to accurately identify anatomical landmarks. Open-source datasets frequently undergo labeling through the utilization of crowdsourcing methodologies. This method is prone to interindividual variability, as the annotation process is often subjective and may differ between individuals [47,48]. Furthermore, most current pose estimation algorithms are trained on 2D video data, which may introduce inaccuracies when estimating joint angles and body segment orientations in 3D space. While some approaches attempt to reconstruct 3D poses, these are often limited in precision compared to marker-based systems, particularly in complex, multiplanar movements or occluded positions [46]. For these reasons, it is not yet possible to completely replace marker-based 3D motion capture in the laboratory with smartphone apps, but they already enable valid testing and thus facilitate the testing of large sample sizes.

As this scoping review did not aim to evaluate the quality or magnitude of validation outcomes, no conclusions regarding the strength of validity across applications can be drawn.

From a methodological perspective, scoping reviews inherently carry limitations, such as potential selection bias and the lack of critical appraisal. Although we aimed to follow rigorous inclusion criteria, the decision to include or exclude studies may have introduced subjective bias. Furthermore, due to the rapidly evolving nature of pose estimation technologies, it is possible that some recently published or ongoing work was not captured at the time of our review.

Looking forward, we recognize that including articles written in multiple languages could enhance the comprehensiveness of future reviews. By broadening the scope to include non-English studies, we can ensure a more inclusive and thorough understanding of the available research on this topic.

### Conclusions

To our knowledge, this is the first scoping review to provide a comprehensive overview of smartphone apps that assess MPS using video-based markerless motion capture. Our findings highlight a growing interest in this cutting-edge field, with a variety of apps already available. These apps demonstrate significant potential across multiple domains, including enhanced monitoring, accurate kinematic measurements, and real-time feedback.

Looking ahead, there is a clear need for the development of more smartphone apps that assess MPS through video-based motion capture. It is crucial that these apps undergo rigorous validation against gold standard systems, such as marker-based 3D motion tracking systems [14,15,49]. Moreover, it is essential to ensure these apps are tested across diverse populations, including children, adolescents, adults, older adults, and individuals with disabilities.

Future research should also explore the integration of these apps with other health monitoring technologies, such as wearable sensors and telehealth platforms, to create comprehensive health management systems. Additionally, leveraging these apps in remote and underserved areas could offer valuable health insights and improve access to medical care. As AI and machine learning continue to advance, the accuracy and functionality of these apps will be further enhanced, solidifying their roles as effective tools for health and performance monitoring.

In summary, video-based markerless motion capture apps on smartphones hold transformative potential for health and performance monitoring. Continued innovation and validation in this field could lead to more accessible, accurate, and comprehensive monitoring systems, thereby facilitating the work of scientists and the development of interventions.

## Supplementary material

10.2196/65474 Multimedia Appendix 1 Search term.

10.2196/65474 Multimedia Appendix 2 Key findings of the included studies.

10.2196/65474 Multimedia Appendix 3 Heatmap of the motor performance skills (MPS) per target population and validated MPS.

10.2196/65474 Multimedia Appendix 4 Heatmap of the algorithms and frameworks used in smartphone motor performance skill applications.

10.2196/65474 Checklist 1 PRISMA-ScR checklist.

## Abbreviations

AI: artificial intelligence
FMT: functional movement test
iNPH: idiopathic normal pressure hydrocephalus
MPS: motor performance skills
PRISMA-ScR: Preferred Reporting Items for Systematic Reviews and Meta-Analysis extension for Scoping Reviews
TDPT-GT: Three-Dimensional Pose Tracker for Gait Test
