# Supplementary material for: Video-Based Motion Capture Smartphone Apps for Testing Human Motor Performance Skills: Scoping Review
Source: JMIR Mhealth Uhealth. 2026 Feb 19;14:e65474. doi: 10.2196/65474 (PMC12919747; doi:10.2196/65474)
Supplement: Multimedia Appendix 1 [file mhealth-v14-e65474-s001.docx]

## Multimedia Appendix 2. Search terms

### PubMed

(("motor performance" OR "motor testing" OR fitness OR "Physical Fitness"[Mesh] OR "motor monitoring" OR "motor assessment" OR talent OR "Motor Skills"[Mesh] OR "Exercise Test"[Mesh] OR "Hand Strength"[Mesh] OR "Leg/physiology"[Mesh] OR "Kinetics"[Mesh] OR "Posture/physiology"[Mesh] OR "Muscle Strength"[Mesh] OR "Movement/physiology"[Mesh] OR "Athletic Performance"[Mesh] OR "task performance" OR "Walk Test"[Mesh] OR "Biomechanical Phenomena"[Mesh] OR "kinematics" OR "motor ability" OR "Physical Endurance"[Mesh] OR "strength" OR "balance" OR "Postural Balance"[Mesh] OR "coordination" OR "cardiorespiratory" OR "Reaction Time/physiology"[Mesh] OR "Range of Motion, Articular"[Mesh] OR "Physical Functional Performance"[Mesh]) AND ("Motion Capture"[Mesh] OR "body segmentation" OR "video-based assessment" OR "automated video analysis" OR "video capture" OR "Machine Learning"[Mesh] OR "video collection" OR "video based" OR "automated video assessment" OR "video collection" OR "Video Recording"[Mesh] OR "sports technology" OR "digital technology")) AND ("Mobile Applications"[Mesh] OR "Smartphone"[Mesh] OR "smartphone based" OR "smartphone application" OR "app" OR "Cell Phone"[Mesh] OR "iphone app" OR "android app")

### Scopus

( TITLE-ABS-KEY ( "motor performance" OR "motor testing" OR fitness OR "Physical Fitness" OR "motor monitoring" OR "motor assessment" OR talent OR "Motor Skills" OR "Exercise Test" OR "Hand Strength" OR leg/physiology OR kinetics OR posture/physiology OR "Muscle Strength" OR movement/physiology OR "Athletic Performance" OR "task performance" OR "Walk Test" OR "Biomechanical Phenomena" OR kinematics OR "motor ability" OR "Physical Endurance" OR strength OR balance OR "Postural Balance" OR coordination OR cardiorespiratory OR "Reaction Time/physiology" OR "Range of Motion, Articular" OR "Physical Functional Performance" ) ) AND ( TITLE-ABS-KEY ( "Motion Capture" OR "body segmentation" OR "video-based assessment" OR "automated video analysis" OR "video capture" OR "Machine Learning" OR "video collection" OR "video based" OR "automated video assessment" OR "video collection" OR "Video Recording" OR "sports technology" OR "digital technology" ) ) AND ( TITLE-ABS-KEY ( "Mobile Applications" OR smartphone OR "smartphone based" OR "smartphone application" OR app OR "Cell Phone" OR "iphone app" OR "android app" ) )

### Web of Science

((TS=(“motor performance" OR "motor testing" OR fitness OR "Physical Fitness" OR "motor monitoring" OR "motor assessment" OR talent OR "Motor Skills" OR "Exercise Test" OR "Hand Strength" OR Leg/physiology OR Kinetics OR Posture/physiology OR "Muscle Strength" OR Movement/physiology OR "Athletic Performance" OR "task performance" OR "Walk Test" OR "Biomechanical Phenomena" OR kinematics OR "motor ability" OR "Physical Endurance" OR strength OR balance OR "Postural Balance" OR coordination OR cardiorespiratory OR "Reaction Time/physiology" OR "Range of Motion, Articular" OR "Physical Functional Performance")) AND TS=("Motion Capture" OR “body segmentation” OR "video-based assessment" OR "automated video analysis" OR "video capture" OR "Machine Learning" OR "video collection" OR "video based" OR "automated video assessment" OR "video collection" OR "Video Recording" OR "sports technology" OR "digital technology")) AND TS=("Mobile Applications" OR Smartphone OR "smartphone based" OR "smartphone application" OR app OR "Cell Phone" OR "iphone app" OR "android app")

### Education Resources Information Centre (ERIC)

abstract:("motor performance" OR "motor testing" OR fitness OR "Physical Fitness" OR "motor monitoring" OR "motor assessment" OR talent OR "Motor Skills" OR "Exercise Test" OR "Hand Strength" OR Leg/physiology OR Kinetics OR Posture/physiology OR "Muscle Strength" OR Movement/physiology OR "Athletic Performance" OR "task performance" OR "Walk Test" OR "Biomechanical Phenomena" OR kinematics OR "motor ability" OR "Physical Endurance" OR strength OR balance OR "Postural Balance" OR coordination OR cardiorespiratory OR "Reaction Time/physiology" OR "Range of Motion, Articular" OR "Physical Functional Performance") AND ("Motion Capture" OR “body segmentation” OR "video-based assessment" OR "automated video analysis" OR "video capture" OR "Machine Learning" OR "video collection" OR "video based" OR "automated video assessment" OR "video collection" OR "Video Recording" OR "sports technology" OR "digital technology") AND ("Mobile Applications" OR Smartphone OR "smartphone based" OR "smartphone application" OR app OR "Cell Phone" OR "iphone app" OR "android app")

### Sagepub

"motor performance" OR "motor testing" OR fitness OR "Physical Fitness" OR "motor monitoring" OR "motor assessment" OR talent OR "Motor Skills" OR "Exercise Test" OR "Hand Strength" OR Leg/physiology OR Kinetics OR Posture/physiology OR "Muscle Strength" OR Movement/physiology OR "Athletic Performance" OR "task performance" OR "Walk Test" OR "Biomechanical Phenomena" OR kinematics OR "motor ability" OR "Physical Endurance" OR strength OR balance OR "Postural Balance" OR coordination OR cardiorespiratory OR "Reaction Time/physiology" OR "Range of Motion, Articular" OR "Physical Functional Performance" AND "Motion Capture" OR “body segmentation” OR "video-based assessment" OR "automated video analysis" OR "video capture" OR "Machine Learning" OR "video collection" OR "video based" OR "automated video assessment" OR "video collection" OR "Video Recording" OR "sports technology" OR "digital technology" AND "Mobile Applications" OR Smartphone OR "smartphone based" OR "smartphone application" OR app OR "Cell Phone" OR "iphone app" OR "android app"

### SPORTDiscus

((("motor performance" OR "motor testing" OR "fitness" OR DE "Physical Fitness" OR "motor monitoring" OR "motor assessment" OR "talent" OR DE "Motor Skills" OR DE "Exercise Test" OR DE "Hand Strength" OR DE "Leg/physiology" OR DE "Kinetics" OR DE "Posture/physiology" OR DE "Muscle Strength" OR DE "Movement/physiology" OR DE "Athletic Performance" OR "task performance" OR DE "Walk Test" OR DE "Biomechanical Phenomena" OR "kinematics" OR "motor ability" OR DE "Physical Endurance" OR "strength" OR "balance" OR DE "Postural Balance" OR "coordination" OR "cardiorespiratory" OR DE "Reaction Time/physiology" OR DE "Range of Motion, Articular" OR DE "Physical Functional Performance") AND (DE "Motion Capture" OR “body segmentation” OR "video-based assessment" OR "automated video analysis" OR "video capture" OR DE "Machine Learning" OR "video collection" OR "video based" OR "automated video assessment" OR "video collection" OR DE "Video Recording" OR "sports technology" OR "digital technology")) AND (DE "Mobile Applications" OR DE "Smartphone" OR "smartphone based" OR "smartphone application" OR "app" OR DE "Cell Phone" OR "iphone app" OR "android app"))
