# Supplementary material for: Video-Based Motion Capture Smartphone Apps for Testing Human Motor Performance Skills: Scoping Review
Source: JMIR Mhealth Uhealth. 2026 Feb 19;14:e65474. doi: 10.2196/65474 (PMC12919747; doi:10.2196/65474)
Supplement: Multimedia Appendix 2 [file mhealth-v14-e65474-s002.docx]

## Multimedia Appendix 5. Key findings of the included studies

| **Study** | **Further informations** |
| --- | --- |
| Aoyagi et al., (2022) [32] | App is able to reconstruct full-body human motion efficiently; statistical analysis: NA |
| Azhand et al., (2021) [37] | Excellent validity (ICC (2, k) = 0.958-0.987) and repeatability (ICC (3, 1) = 0.915-0.950) for all measured parameters |
| Balsalobre-Fernández, (2024) [38] | Very high validity between app and force plate (r = 0.971, 95% CI = 0.963-.975); Very high reliability (ICC = 0.969, 95% CI = 0.963-0.975) |
| Fanton et al., (2022) [39] | Significant correlation between Halo Movement score and functional movement tests (r = 0.29-0.63); significant correlations between app and sensor metrics (r = 0.23-0.83) |
| Feng et al.,(2022) [40] | App is capable to capture the movement state accurately, locate the incorrect posture of the swimmer and provide feedback; statistical analysis: NA |
| Iseki et al., (2023) [35] | App detects impaired fluctuations in movements in trunk and limb body positions in iNPH and PD patients; statistical analysis: one-way ANOVA (no F-values were reported) |
| Iseki et al., (2023) [34] | High accuracy in distinguishing pathological gait patterns from healthy gait patterns (AUC = 0.719) |
| Van den Hoorn et al., (2024) [36] | Strong relation between app and 3D motion capture (R^2^ > 0.92); Shoulder ROM was consistently overestimated by the app |
| Yamada et al., (2023) [33] | Indices shuffling gate: hip joint angle < 30° (AUC 77.1, 95% CI = 72.0-82.2), knee joint <45° (AUC 78.6, 95% CI = 73.7-83.4), heel amplitude <0.1 (AUC 71.7, 95% CI = 66.3-77.2)  Indices short-stepped gait: knee joint angle <45° (AUC 81.7, 95% CI = 77.4-86.0)  Indices wide-based gait: leg outward shift >0.1 (AUC 80.6, 95% CI = 76.4-84.8)  App can detect pathological gait features; enables non-expert operation |
| Young et al., (2023) [21] | Good to excellent agreement between app and 3D motion capture (ICC (2, 1) = 0.751-0.981); discrepancies in right foot outcome |

Abbreviation: AUC, area under the curve; ICC, Intra-class correlation
