# Supplementary figures and images for: Video-Based Motion Capture Smartphone Apps for Testing Human Motor Performance Skills: Scoping Review
Source: JMIR Mhealth Uhealth. 2026 Feb 19;14:e65474. doi: 10.2196/65474 (PMC12919747; doi:10.2196/65474)

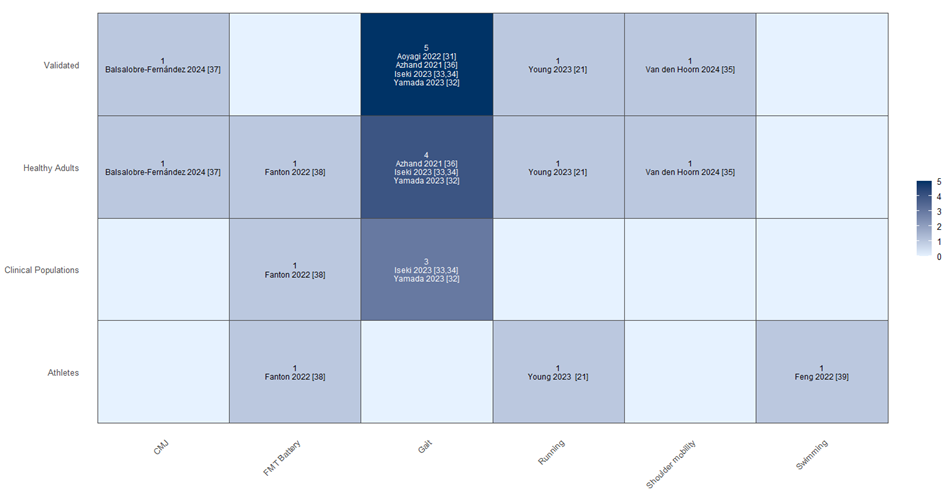

Supplement: Multimedia Appendix 3 [file mhealth-v14-e65474-s003.png]

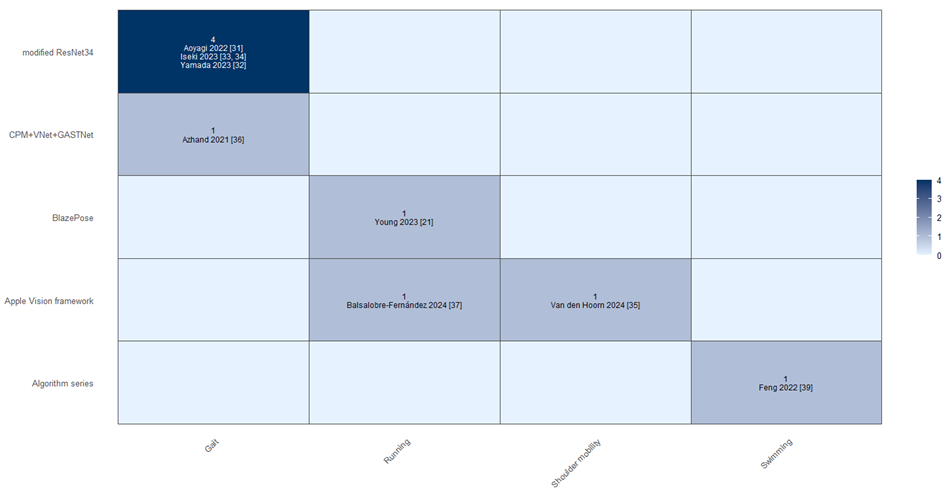

Supplement: Multimedia Appendix 4 [file mhealth-v14-e65474-s004.png]
